# Anti-diabetic effects of Dachaihu decoction combined with *Sanghuangporus vaninii*: insights from network pharmacology, intestinal flora, and liver metabolomics

**DOI:** 10.3389/fcell.2025.1707392

**Published:** 2025-11-10

**Authors:** Juntao Huang, Yanfa Lin, Zifeng Huang, Tiantian Li, Xiaodong Ge, Bin Liu, Zirui Huang

**Affiliations:** 1 College of Food Science, Fujian Agriculture and Forestry University, Fuzhou, China; 2 National Engineering Research Center of JUNCAO Technology, Fujian Agriculture and Forestry University, Fuzhou, China; 3 Fuzhou Institute of Oceanography, Minjiang University, Fuzhou, China; 4 College of Marine and Bioengineering, Yancheng Institute of Technology, Yancheng, China

**Keywords:** Dachaihu decoction, *Sanghuangporus vaninii*, anti-diabetic activity, intestinal flora, liver metabolomics

## Abstract

Numerous studies have demonstrated the therapeutic effects of the traditional Chinese medicinal fungus *Sanghuangporus vaninii* on type 2 diabetes mellitus (T2DM), yet the underlying mechanisms remain unclear. This study aimed to investigate the anti-diabetic and hypolipidemic effects of the *S. vaninii* combined with Dachaihu decoction formulation (DCHD-SV) in T2DM mice and deeply explore its action mechanisms through network pharmacology, 16S RNA sequencing, and liver metabolomics. The results showed that DCHD-SV can effectively ameliorate relevant symptoms of T2DM in mice, including hyperglycemia, dyslipidemia, insulin resistance, and organ damage. In addition, DCHD-SV modulates the intestinal flora and liver metabolites of T2DM mice, and may exert its anti-diabetic effects through the PPARγ/AMPK/mTOR signaling pathway. These findings underscore DCHD-SV as a promising candidate for T2DM therapy and lay a theoretical foundation for further investigating its mechanisms.

## Graphical Abstract

**Figure:**
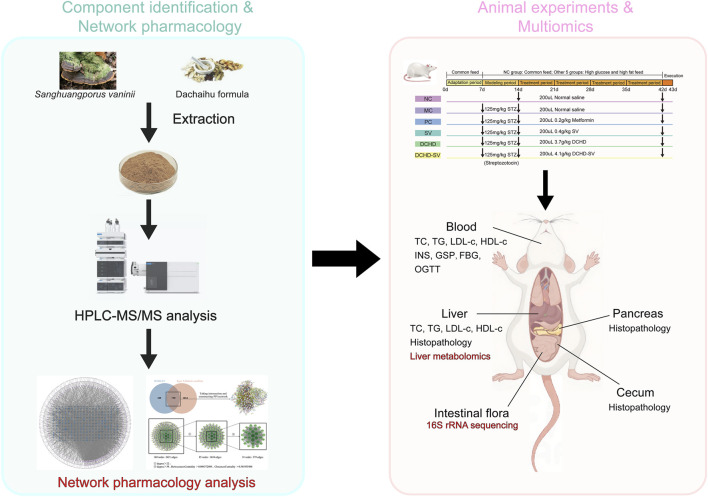

## 1 Introduction

Diabetes mellitus (DM) is a metabolic disorder characterized by hyperglycemia resulting from defective insulin secretion or impaired insulin action. It induces to complications across multiple systems, including cardiovascular, retinal, and renal pathologies (Iatcu et al., 2022).

In recent years, many natural herbal formulas and extracts have been found to have anti-diabetic properties, which have received extensive attention due to their stable efficacy and low side effects (Xu et al., 2018). Dachaihu decoction (DCHD) is a well-known traditional Chinese medicine formula that has been used to treat type 2 diabetes mellitus (T2DM) (Zhang et al., 2022). The ingredients of DCHD include multiple herbs with potential anti-diabetic effects, such as *Bupleurum chinense* (Feng et al., 2019), *Scutellaria baicalensis* (Xiao S. W. et al., 2020), *Rheum officinale* (Gao et al., 2010), *Paeonia lactiflora* (Yang et al., 2016), and *Zingiber officinale* (Arablou et al., 2014).

*Sanghuangporus* (Sanghuang) is a traditional Chinese medicinal fungus with a medicinal history of over 2,000 years. Modern scientific studies have shown that *Sanghuangporus* contains multiple classes of active substances and exhibits broad biological activities (Lin et al., 2023). In previous studies, we have confirmed the hypoglycemic and hypolipidemic activity of water (SVW) and ethanol extracts (SVE) of *S*. *vaninii* (Huang et al., 2022a), as well as their anti-diabetic effects in combination with other traditional Chinese medicines (Huang et al., 2024).

In this study, we further combined SV (SVW & SVE) with DCHD to develop a novel formulation (DCHD-SV), and investigated its hypoglycemic and hypolipidemic activity in T2DM mice. At the same time, we also investigated its effect on the intestinal flora and liver metabolomics of T2DM mice to explore the potential mechanism of its action. Our research provides a theory basis for the treatment of T2DM based on the intestinal-hepatic axis.

## 2 Materials and methods

### 2.1 Materials and reagents

The fruiting body of *S*. *vaninii* was obtained from Hangzhou Academy of Agricultural Sciences (Hanzhou, China). Chinese herbal pieces purchased from Sichuan Guoqiang Chinese Herbal Pieces Co., Ltd. (Chengdu, China). Acarbose was provided by Saen Chemical Technology Co., Ltd. (Shanghai, China). Anhydrous ethanol and anhydrous sodium carbonate were from Sinopharm Chemical Reagent Co., Ltd. (Shanghai, China). Metformin hydrochloride, streptozotocin (STZ), α-glucosidase, p-nitrophenyl-α-D-glucopyranoside (p-NPG) and saline solution were provided by Shanghai Yuanye Bio-Technology Co., Ltd. (Shanghai, China). Maintenance feed and high-sugar and high-fat feed were provided by Beijing Huafukang Biotechnology Co., Ltd. (Beijing, China) and Wu’s animal experimental center (Fuzhou, China), respectively.

### 2.2 Preparation of SV, DCHD and DCHD-SV

**FIGURE 1:**
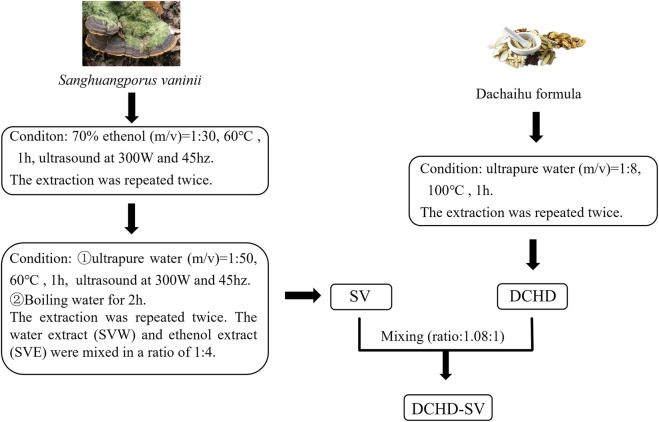
The preparation process of SV, DCHD and DCHD-SV.

The preparation methods of SVW and SVE refer to the previous studies in our laboratory (Huang et al., 2024), and the preparation of DCHD is based on the solubility of the main active ingredients of medicine herbs. The specific preparation process is shown in the Figure 1, SV and DCHD were prepared according to fixed protocols, with raw material screening conducted in accordance with the Chinese Pharmacopoeia.

### 2.3 UPLC-MS/MS analysis and network pharmacology analysis of DCHD-SV

The main compounds of DCHD-SV were determined by HPLC-MS/MS refer to a pervious study (Huang et al., 2023). The raw mass spectrometry data were format-converted and processed using Proteowizard (v3.0.8789) and R XCMS (v3.12.0) to obtain quantitative information of substances, including mass-to-charge ratio (mz) and retention time (rt). Subsequently, data normalization was performed using the total peak area normalization method (Navarro-Reig et al., 2015). Finally, qualitative results of DCHD-SV components were obtained by searching databases such as HMDB, MassBank, LIPID MAPS, mzCloud, and KEGG, followed by comparison with mass spectrometry data.

The DCHD-SV components detected by UPLC-MS/MS were input into SwissADME for pharmacokinetic evaluation and screening. After screening, the effective components were predicted by Swiss TargetPredition to obtaine the component targets. The targets related to T2DM were obtained from Genecards, DrugBank and OMIM databases, and the cross analysis between component targets and T2DM-related targets was carried out to find out their common targets. Then, Protein-Protein Interaction (PPI) network analysis was constructed by crossing targets to screen key targets by STRING. Finally, GO function and KEGG pathway enrichment were analyzed for the key targets, and visual analysis was performed using SR POLT platform (Tang et al., 2023).

### 2.4 The α-glucosidase inhibitory activity *in vitro*

The α-glucosidase inhibitory activity *in vitro* was determined with reference to a previous report (Ma et al., 2008). The enzymatic reaction was performed by sequentially adding 80 μL of p-nitrophenyl-α-D-glucopyranoside (pNPG, 5 mM) and 10 μL of serially diluted samples (0.001, 0.01, 0.1, 1, and 10 mg/mL) to a 96-well plate. The mixture was incubated at 37 °C for 5 min before adding 10 μL of α-glucosidase solution (0.2 U/mL). After further incubation at 37 °C for 15 min, the reaction was terminated with 100 μL of sodium carbonate (0.2 M). The absorbance was subsequently measured at 405 nm using a microplate reader. The α-glucosidase inhibitory activity was calculated with PBS substituting for the sample or α-glucosidase as the control or background group using the following formula:

The inhibition activity α-glucosidase (%) = [1-(A_1_-A_2_)/(A_3_-A_4_)] × 100%

A_1_ – Absorbance of the sample group.

A_2_ – Absorbance of the sample background group.

A_3_ – Absorbance of the control group.

A_4_ – Absorbance of the control background group.

### 2.5 Animal experiment

The 36 male ICR mice (SPF, 6 weeks age, 26 ± 2 g) were supplied by Wu Shi’s Experimental Center Laboratory (Fuzhou, China). The mice were housed in an environmentally controlled space with 23 ± 2 °C, 55% ± 5% relative humidity, and a 12/12 light-dark cycle.

Following the adaptation phase, 6 mice were randomly assigned as normal control (NC) group and continued to be fed with maintenance diet. After fasting for 8–12 h, the remaining 30 mice were intraperitoneally injected with STZ solution at a dose of 125 mg/kg and fed on a high-sugar, high-fat diet for the following week. After 1 week, the mice were fasted for 8–12 h, and the fasting blood glucose (FBG) was measured. Then, these 30 mice were randomly divided into the following 5 groups: model control (MC) group, positive control (PC) group, *S. vaninii* (SV) group, DCHD group, and DCHD-SV group. The mice in each group were administered drugs via gavage for 4 weeks, and the specific gavage samples and dosages referred to Figure 3A.

### 2.6 Sampling and determination

Following successful modeling and grouping, the initial fasting blood glucose (FBG_0_) and body weight (BW_0_) were recorded. FBG and BW of mice were measured every 2 weeks during intragastric administration. After 4 weeks, an oral glucose tolerance test (OGTT) was conducted. Following an 8–12 h fasting period with *ad libitum* water access, FBG were measured and designated as G_0_. Mice then received a 2 g/kg glucose solution, and serial blood glucose levels were determined at 0.5 h (G_0.5_), 1.0 h (G_1.0_), and 2.0 h (G_2.0_) post-administration. The area under the curve (AUC) of OGTT was calculated using the corresponding formula. Subsequently, all mice were fasted for 12 h, and blood was taken from the eyeballs. The mice were euthanized by cervical dislocation, and the liver, pancreas, cecum, and intestinal contents were dissected. The collected blood was left for 30 min, and the serum was obtained by centrifugation and stored at −20 °C. The liver tissue was fixed in 4% paraformaldehyde solution and kept at room temperature and other tissues were frozen in liquid nitrogen and stored at −80 °C.

Glycated serum protein (GSP), total cholesterol (TC), triglyceride cholesterol (TG), high-density lipoprotein cholesterol (HDL-c) and low-density lipoprotein cholesterol (LDL-c) were quantified by the kits (Jiancheng, Nanjing, China). Insulin releasing test (INS) in the serum were quantified by ELISA kits (Purity Biotechnology, Wuhan, China). The homeostasis model assessment insulin resistance index (HOMA-IRI), β-cell function (HOMA-β), insulin sensitivity index (HOMA-ISI) and quantitative insulin sensitivity check index (QUICKI) were assessed using the following formulas.

$$\text{AUC}=\left(G_{0}+G_{0.5}\right)\times 0.25+\left(G_{0.5}+G_{1.0}\right)\times 0.25+\left(G_{1.0}+G_{2.0}\right)\times 0.5$$

$$\text{HOMA}-\text{IRI}=\left(\text{FBG}\times \text{INS}\right)/22.5$$

$$\text{HOMA}-\beta =20\times \text{INS}/\left(\text{FBG}-3.5\right)$$

$$\text{HOMA}-\text{ISI}=22.5/\left(\text{FBG}\times \text{INS}\right)$$

$$\text{QUICKI}=1/\left(\text{lgINS}+\text{lgFBG}\right)$$

### 2.7 Assessment of histopathology

The liver, cecum and pancreas tissues were fixed, embedded in paraffin, sectioned, and stained with hematoxylin and eosin. Then, the section morphology was observed by optical microscope (Huang et al., 2024).

### 2.8 Analysis of intestinal flora

The 16S rRNA sequencing was performed at Shanghai Personal Biotechnology Co., Ltd. (Shanghai, China), and the specific methods can be referred to our previous paper (Huang et al., 2022b). The Alpha diversity index was calculated by QIIME 2 software, and the Beta diversity of intestinal flora was analyzed by SIMCA software, and the principal coordinates analysis (PCoA) chart was drawn. The relative abundance of intestinal microorganisms was analyzed for differences between groups, combined with LDA Effect Size (LEfSe) analysis (LDA>2.0, *p* < 0.05) to find differential flora. The Spearman correlation analysis was performed on the differential flora and biochemical indicators through the Lianchuan BioCloud platform, and the data with |r| > 0.5 and *p* < 0.05 were selected to draw a network diagram.

### 2.9 Analysis of liver metabolites

Liver metabolite profiling was conducted by Suzhou Panomix Biomedical Tech Co., Ltd. (Suzhou, China). The metabolome data were analyzed using SIMCA software with principal component analysis (PCA), partial least squares-discriminant analysis (PLS-DA), and orthogonal partial least squares-discriminant analysis (OPLS-DA), and permutation tests were used to evaluate model reliability. Secondary mass spectrometry results were matched against HMDB, Massbank, LIPID MAPS, mzCloud, and KEGG databases. The pathway enrichment analysis of differential metabolites between each group and the MC group was conducted using MetaboAnalyst 4.0, followed by hierarchical clustering heatmap visualization. Spearman’s correlation analysis among biomarkers, intestinal flora, and biochemical indicators was performed via the Lianchuan BioCloud platform, with network diagrams constructed using data meeting |r| > 0.5 and *p* < 0.05 thresholds (Lyu et al., 2023).

### 2.10 Statistics

All data were exhibited as mean ± standard deviation (SD), and Graphpad prism (ver. 9.0) was conducted to one-way ANOVA analysis. Tukey’s test was applied for multiple comparisons, and the significance of differences between groups was described as *p < 0.05, **p < 0.01, and ***p < 0.001.

## 3 Results and discussion

### 3.1 The main compounds and network pharmacology analysis of DCHD-SV

The secondary mass spectrometry results of DCHD-SV were matched with the database, and 2,447 compounds were detected. The main compounds of DCHD-SV are shown in Table 1, in which oroxylin A-7-o-β-D-glucuronide is one of the major components of DCHD-SV. The present studies have found that oroxylin A-7-o-β-D-glucuronide has a certain hypoglycemic potential, which can improve the epithelial mesenchymal transition injury in diabetic nephropathy (Xie et al., 2024). Baicalin is also one of main components of DCHD-SV, which can improve diabetic neuropathy and kidney histopathology (Ma et al., 2021). In addition, DCHD-SV is also rich in stachydrine, which has been shown to alleviate inflammation and promote autophagy in diabetic retinopathy (Yu et al., 2023). The results of component identification showed that DCHD-SV contains multiple anti-diabetic active components and is worthy of further investigation.

**TABLE 1:** The main compounds of DCHD-SV by using UPLC-MS/MS.

KEGG	Name	Formula	Classification
NULL	Oroxylin A-7-o-β-D-glucuronide	C_22_H_20_O_11_	NULL
C10112	Hexamethylquercetagetin	C_21_H_22_O_8_	Flavonoids
C10025	Baicalin	C_21_H_18_O_11_	Flavonoids
C06165	Macarpine	C_22_H_18_NO_6_^+^	Isoquinoline alkaloids
C14472	Zapotin	C_19_H_18_O_6_	Flavonoids
C19565	4-Hydroxy-1-(3-pyridinyl)-1-butanone	C_9_H_11_NO_2_	Aromatic ketone
C10172	Stachydrine	C_7_H_13_NO_2_	Pyrrolidine alkaloids
C02495	3'-Hydroxydaidzein	C_15_H_10_O_5_	Isoflavonoids
C00148	L-Proline	C_5_H_9_NO_2_	Amino acids
C00123	L-Leucine	C_6_H_13_NO_2_	Amino acids
C19582	1-(3-Pyridinyl)-1,4-butanediol	C_9_H_13_NO_2_	Pyridines
C00780	Serotonin	C_10_H_12_N_2_O	Indole alkaloids
C18910	2,5-diamino-6-(5-phospho-D-ribitylamino)pyrimidin-4(3H)-one	C_9_H_18_N_5_O_8_P	Glycosides
C12135	Dehydroferreirin	C_16_H_12_O_6_	Isoflavones
C04712	(7R)-7-(5-Carboxy-5-oxopentanoyl)aminocephalosporinate	C_16_H_18_N_2_O_9_S	Lactams
C07911	phenylpropanolamine	C_9_H_13_NO	Benzoic acids

**FIGURE 2:**
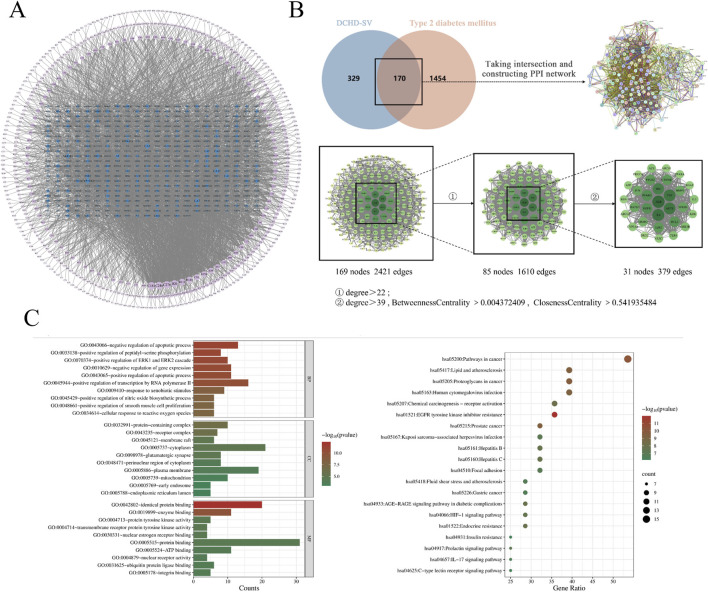
The network pharmacology of DCHD-SV on T2DM. Compound-target network of DCHD-SV **(A)**, intersection targets and core targets screening of DCHD-SV and T2DM **(B)**, GO and KEGG pathway analysis at core targets of DCHD-SV and T2DM **(C)**.

The action mechanism of traditional Chinese medicine has the characteristics of multi-target and multi-level, which is similar to the integrity, systematization and comprehensiveness of network pharmacology. Therefore, network pharmacology is highly suitable for the pharmacological research of Chinese medicine formula (Zhao et al., 2023). As shown in Figure 2A, the components of DCHD-SV were analyzed and screened for 499 targets, and a component-target network of DCHD-SV was constructed. As shown in Figure 2B, component targets were intersected with disease targets of T2DM and PPI network analysis was constructed to screen out 31 key targets, including MAPK3, PPARγ, NFKB1 and AKT1. As one of the main active compounds of DCHD-SV, baicalin participates in the regulation of glucose and lipid metabolism homeostasis by activating and upregulating AMPK and PPARγ (Rahimi et al., 2021). Additionally, fluoxetine hydrochloride, a selective serotonin reuptake inhibitor, may regulate glucose and lipid metabolism via the PI3K-AKT signaling pathway in diabetic rats (Yang et al., 2020). Meanwhile, the 20 results of pathways from GO and KEGG enrichment analysis are shown in Figure 2C, and the important diabetes-related KEGG signaling pathways of key target genes was shown in Supplementary Table S1, including lipid and atherosclerosis, chemical carcinogenesis - receptor activation, fluid shear stress and atherosclerosis, HIF-1 signaling pathway, insulin resistance, IL-17 signaling pathway, C-type lectin receptor signaling pathway. Results from network pharmacology preliminarily indicate that DCHD-SV may have potential anti-diabetic efficacy; thus, we conducted further experiments to investigate this.

### 3.2 Effect of α-glucosidase inhibitory activity of DCHD-SV

**FIGURE 3:**
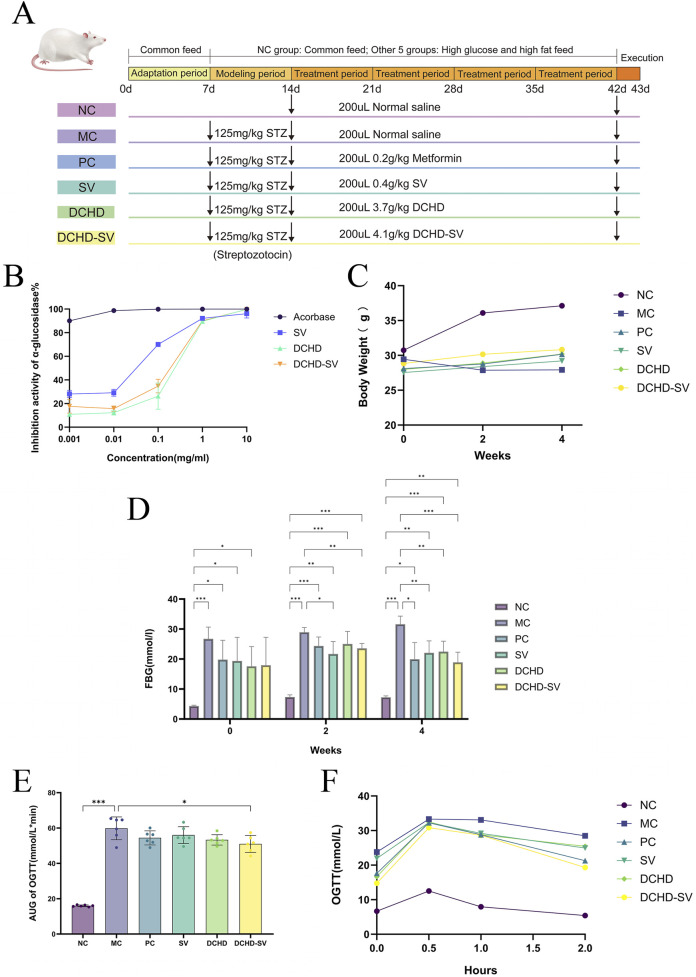
The schematic diagram of animal experiment **(A)**. The α-glucosidase inhibitory activity of SV, DCHD, DCHD-SV *in vitro* **(B)** (*n* = 3). Effects of SV, DCHD, DCHD-SV administration on the levels of BW **(C)**, FBG **(D)**, AUC of OTGG **(E)**, and OTGG **(F)** (*n* = 6). Note: *p < 0.05, **p < 0.01, ***p < 0.001.

The α-glucosidase inhibitors can inhibit the decomposition of disaccharides and oligosaccharides and the release of α-D glucose, thus delaying the absorption of glucose in the small intestine and effectively reducing postprandial hyperglycemia and its harmful physiological disorders (Hossain et al., 2020). As shown in Figure 3B, acarbose is an excellent inhibitor of α-glucosidase at low concentrations. The α-glucosidase inhibitory activity of DCHD and DCHD-SV increased rapidly at concentrations of 0.1–10 mg/mL, the α-glucosidase inhibitory activities of DCHD and DCHD-SV were 89.57% ± 0.93% and 90.07% ± 0.75%, respectively. The IC_50_ of DCHD and DCHD-SV were 0.2782 mg/mL and 0.2434 mg/mL, respectively. Numerous studies have demonstrated that extracts and active components of *Sanghuangporus* exhibit favorable glucosidase inhibitory activity *in vitro* (Cheng et al., 2020; Wang et al., 2025). From the results of this experiment, DCHD-SV has a better performance than DCHD, but still need to be further explored through animal experiments.

### 3.3 DCHD-SV ameliorates biochemical indicators in T2DM mice

#### 3.3.1 Effect of DCHD-SV on BW and FBG

High-sugar and high-fat diet can make mice gain weight, induce insulin resistance and increase the risk of T2DM (Gheibi et al., 2017). As shown in Figure 3C, at the time of successful modeling (week 0), mice in NC group were significantly heavier than the other diabetic mice. As the experiment went on, each administration group showed a tendency to mitigate weight loss in mice by the 2nd week. By week 4, with the progression of T2DM, the BW of mice in the MC group was significantly lower than that of normal healthy mice (*p* < 0.001); in contrast, BW in each administration group was significantly higher than that in the MC group (*p* < 0.001), but still lower than that in the NC group. These results showed that the weight loss of T2DM mice could be reversed by drug administration.

As shown in Figure 3D, the FBG of mice in MC group at week 0 was significantly higher than that in NC group (*p* < 0.001). After 4 weeks of administration intervention, the FBG of mice in each administration groups showed a downward trend and was significantly lower than that in MC group. Among them, the improvement effect of DCHD-SV group (*p* < 0.001) on FBG was significantly higher than that of SV (*p* < 0.01) and DCHD (*p* < 0.01). The results are similar to our previous study, which showed that SV combined with other traditional Chinese medicinal herbs had better effect in reducing FBG levels in T2DM mice (Huang et al., 2024). These results indicated that DCHD-SV could improve the weight loss and FBG elevation of T2DM mice.

#### 3.3.2 Effect of DCHD-SV on OGTT, GSP, HOMA-IRI, HOMA-ISI, HOMA-β and QUICKI

OGTT can be used to diagnose impaired glucose tolerance or T2DM in individuals and to study glucose utilization and insulin sensitivity in animals (Kuo et al., 2021). As shown in Figure 3E, the OGTT levels of mice in each group increased sharply after glucose administration, peaked within 0.5 h, then gradually declined and returned to similar levels after 2 h. In addition, the OGTT levels of the MC group were significantly higher than those in the NC group at all time points, with levels in other administration groups intermediate between the two. This indicates that the development of T2DM impaired glucose tolerance impairment in mice, with partial recovery after drug administration. As shown in Figure 3F, the area under the curve (AUC) of OGTT in each drug administered group decreased, but only the DCHD-SV group showed a statistically significant difference (*p* < 0.01). The results indicated that DCHD-SV could effectively improve the glucose tolerance injury caused by T2DM in mice.

**FIGURE 4:**
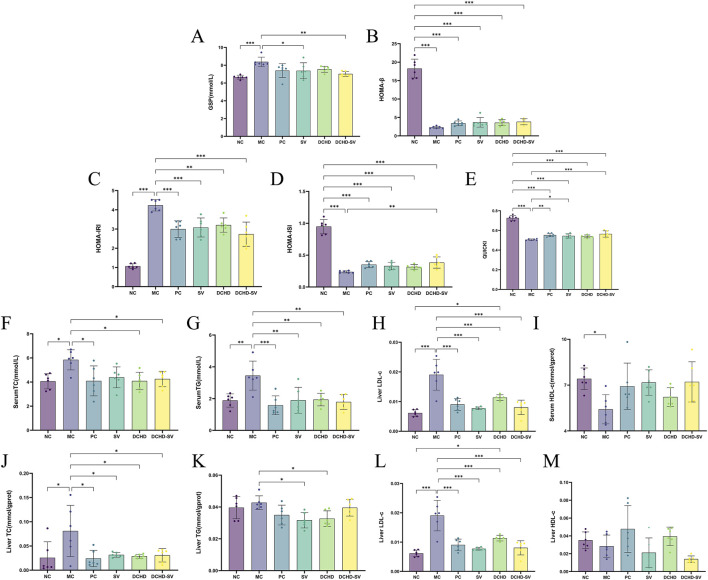
Effects of SV, DCHD, DCHD-SV administration on the levels of GSP **(A)**, HOMA-β **(B)**, HOMA-IRI **(C)**, HOMA-ISI **(D)**, QUICKI **(E)**, serum TC **(F)**, serum TG **(G)**, serum LDL-c **(H)**, serum HDL-c **(I)**, liver TC **(J)**, liver TG **(K)**, liver LDL-c **(L)** and liver HDL-c **(M)** in T2DM mice (*n* = 6). Note: *p < 0.05, **p < 0.01, ***p < 0.001.

GSP is a product of glycation reaction between glucose and serum proteins in the blood circulation, which can be used alone or in combination with glycated hemoglobin for the screening and diagnosis of DM (Wang et al., 2019). As shown in Figure 4A, the GSP levels in the MC group was significantly higher than that in the NC group (*p* < 0.001). After 4 weeks of intervention, the GSP levels in SV, and DCHD-SV groups were significantly lower than that in the MC group. Among them, the improvement effect of the DCHD-SV group (*p* < 0.01) on GSP was better than that of SV group (*p* < 0.05), indicating that DCHD-SV can effectively improve the abnormal GSP level caused by T2DM.

Indirect indices of insulin resistance serve to evaluate not only cellular resistance to insulin but also pancreatic beta-cell function and insulin sensitivity (Placzkowska et al., 2019). The study utilized four insulin-related indexes to evaluate insulin resistance and pancreatic β-cell function in each group of mice (Figures 4B–E). The results showed that the HOMA-IRI of mice in the MC group was significantly higher than that in the NC group (*p* < 0.001), while the HOMA-ISI, HOMA-β, and QUICKI were significantly lower than those in the NC group (*p* < 0.001). Notably, all administration groups showed improvements to varying degrees in these indicators, and DCHD-SV demonstrated the best performance among all administration groups in each indicator. Compared with the MC group, DCHD-SV decreased HOMA-IRI by 35.52% (*p* < 0.001), and increased HOMA-ISI, HOMA-β, and QUICKI by 61.52% (*p* < 0.01), 67.67%, and 11.49% (*p* < 0.001), respectively. The reduction in HOMA-IRI reflected that DCHD-SV alleviated insulin resistance in T2DM mice (Tahapary et al., 2022), whereas the increases in HOMA-ISI and QUICKI indicated that DCHD-SV enhanced insulin sensitivity in these mice. Regarding HOMA-β, DCHD-SV showed a certain degree of improvement without statistical significance, and the levels remained much lower than that in the NC group. This may be attributed to the fact that STZ impairs the function of β-cells in mice, and the persistent hyperglycemia induced by STZ inhibits the recovery of β-cell function through multiple mechanisms (Hahn et al., 2020). These results indicate that DCHD-SV exerts a favorable effect on alleviating insulin resistance and enhancing insulin sensitivity in T2DM mice.

#### 3.3.3 Effect of DCHD-SV on hyperlipidemia

Dyslipidaemia is defined as elevated levels of plasma triacylglycerols and cholesteryl esters, which are components of LDL-c and HDL-c (Eid et al., 2019). In this study, serum TC, TG, LDL-c and HDL-c were measured to investigate the effect of SV, DCHD, DCHD-SV on hyperlipidemia in T2DM mice (Figures 4F–I). It can be found that the levels of TC, TG, and LDL-c in the MC group were significantly higher than those in the NC group, with increases of 43.80% (*p* < 0.05), 83.12% (*p* < 0.01), and 61.99% (p < 0.01), respectively. By contrast, the HDL-c level was significantly decreased by 26.98% (*p* < 0.05). After 4 weeks of intervention, varying degrees of improvement in dyslipidemia were observed in all administration groups. Collectively, the levels of TC, TG, and LDL-c in the SV, DCHD, and DCHD-SV groups were all significantly lower than those in the MC group. However, the DCHD group showed a relatively limited effect for the elevation of HDL-c levels, while the SV and DCHD-SV groups exhibited more effective with increases of 32.37% and 33.13%. Specifically, at TG and HDL-c levels, the DCHD-SV group performed the best of the three groups. In contrast, for TC and LDL-c levels, this group performed slightly less favorably than the DCHD group but remained superior to the SV group. Heretofore, studies have shown that DCHD can regulate the levels of TC, TG, HDL-c and LDL-c in T2DM mice (Shi et al., 2024), while in our previous research, it was confirmed that SV can also improve dyslipidemia in T2DM mice (Huang et al., 2022a). In this study, we further found that there may be a synergistic effect between the two in reducing TG and increasing HDL-c in T2DM mice. DCHD-SV can exert a more balanced and comprehensive effect in the treatment of dyslipidemia in T2DM mice.

Insulin resistance and T2DM affects liver pathology, typically leading to nonalcoholic fatty liver disease by dynamically altering the hepatic genes involved in glucose and lipid metabolism (Takamura et al., 2012). After intervention, the levels of TC and LDL-c in liver were significantly decreased in all groups compared with those in MC group (Figures 4J,L). In terms of liver TG levels, the SV and DCHD groups demonstrated superior efficacy, with reductions of 25.80% (*p* < 0.05) and 23.41% (*p* < 0.05), respectively (Figure 4K). However, in terms of liver HDL-c, all administration groups failed to show a significant difference compared with the MC group (Figure 4M). These results indicate that each administration group can ameliorate the abnormal hepatic lipid metabolism induced by T2DM in mice to varying degrees.

### 3.4 Effects of DCHD-SV on liver, pancreas and cecum

**FIGURE 5:**
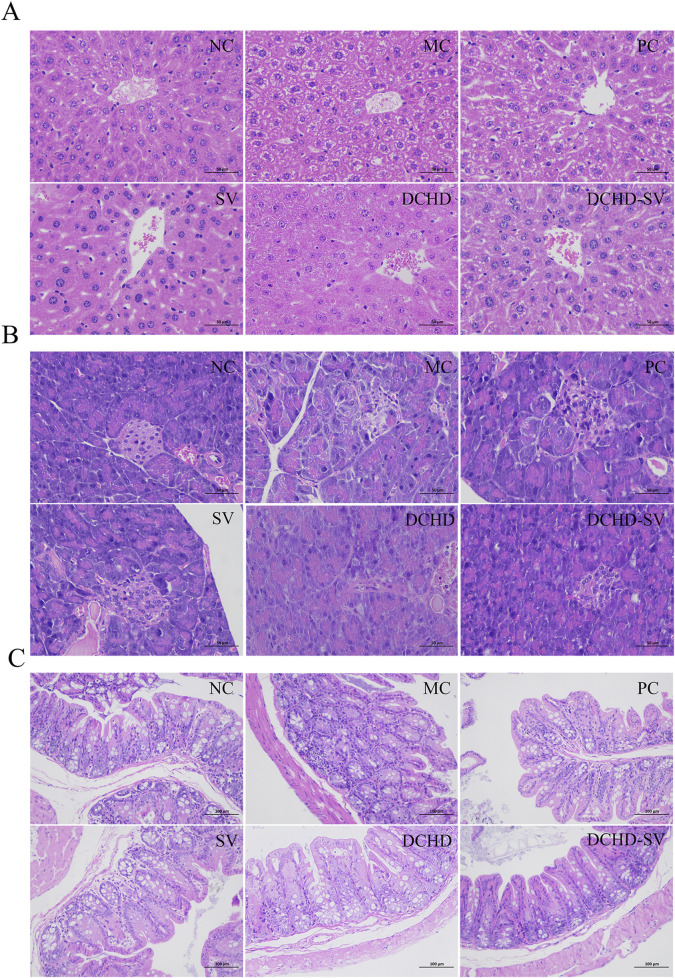
The liver **(A)**, pancreas **(B)** and cecum **(C)** sections by hematoxylin and eosin staining.

The improvement effect of SV, DCHD, DCHD-SV on the liver fat accumulation and injury of T2DM mice was further verified by histological sections (Figure 5A). Compared with the NC group, hepatocytes in the MC group were significantly hypertrophy with lipid vacuoles, and the cell membrane boundaries were unclear, showing obvious hepatocyte damage. Following intervention by different administration groups, the liver tissue morphology in T2DM mice showed partial recovery, with significantly restored hepatocyte volume, regular cellular arrangement, and and a notable decrease in lipid vacuoles. Previous studies have confirmed that DCHD can alleviate hepatic lipid accumulation and has been used as an adjuvant therapy for non-alcoholic fatty liver disease (Mou et al., 2024). Furthermore, our study further revealed that DCHD-SV formulation exerted a unique effect on liver protection in T2DM mice.

The pancreas and cecum histopathology sections of each group of mice (Figures 5B,C). Compared with the NC group, pancreatic islet β cells of T2DM mice in the MC group exhibited morphological alterations, with unclear islet boundaries and disordered structures. Furthermore, the mucosal structure of the MC group was disrupted, and the villi were arranged in a disordered manner, indicating the presence of cecal inflammation. After 4 weeks of intervention, pathological damage to pancreatic islets in mice from each administration group was significantly alleviated, with islet cells regaining ordered and boundaries becoming clearer. Moreover, cecal damage in all administration groups was also alleviated, with the cecal morphology of the DCHD-SV group being the closest to normal. Similarly, a recent study demonstrated that administration of functional formulations of *Chlorella pyrenoidosa* for 4 weeks ameliorated tissue damage in the liver, pancreas, and cecum of STZ-induced T2DM mice (Huang et al., 2023). The histomorphological studies demonstrated the damage induced by STZ in mice and highlighted the ameliorative effect of drug administration intervention on organ inflammation and destruction in T2DM mice.

### 3.5 Effects of DCHD-SV on intestinal flora

**FIGURE 6:**
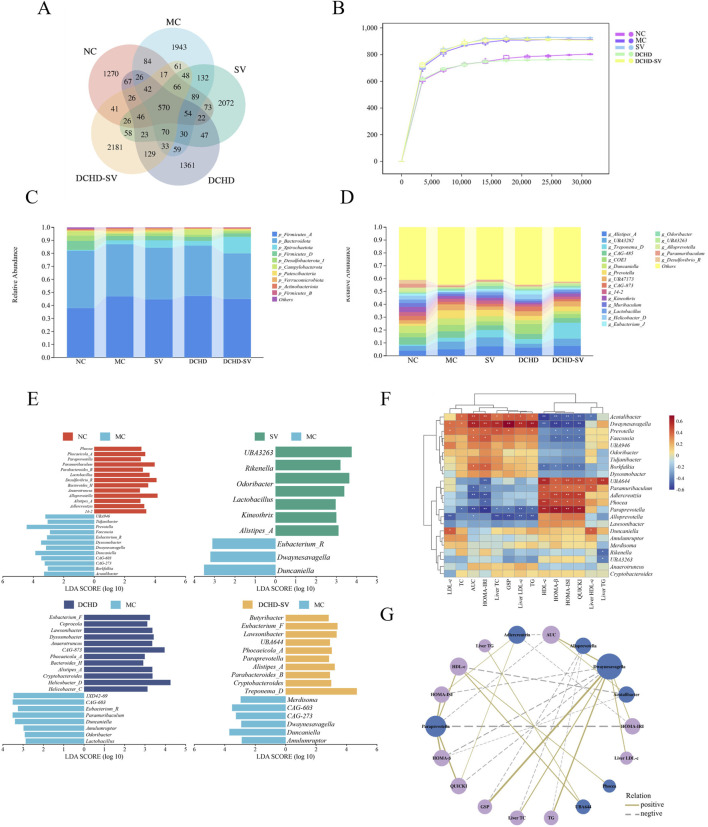
Effects of SV, DCHD, DCHD-SV administration on intestinal flora (*n* = 6). The statistical analysis of ASV **(A)** and rarefaction curve **(B)**, compositions of intestinal flora at phylum level **(C)**, and genus level (LDA>3.0, *p* < 0.05) **(D)** in each group. The LEfSe analysis of intestinal flora comparisons between different groups at the genus level **(E)**. The heatmap of Spearman’s correlation analysis between the intestinal flora and biochemical indexes **(F)**, and network plot **(G)** based on the correlation.

The intestinal flora plays an important role in metabolism and immune regulation. The intestinal flora disturbances combined with the destruction of intestinal barrier in patients with T2DM can damage many organs (Yang et al., 2021). As shown in Figure 6A, the sparse curves of the groups tended to flatten, indicating that the sequencing results were sufficient to reflect the species diversity of the samples. Figure 6B shows Venn diagram of the microorganisms in each group, indicating that SV, DCHD, and DCHD-SV can affect the composition of the intestinal flora in T2DM mice. At the phylum level (Figure 6C), the Firmicutes/Bacteroidetes ratio (F/B value) was higher in the MC group compared with the NC group; in contrast, the F/B values in the SV and DCHD-SV groups were lower than those in the MC group. Additionally, Actinobacteriota was significantly decreased in T2DM mice compared with the NC group, and this phylum includes *Bifidobacterium*, which helps improve digestive issues, regulate blood glucose and lipid levels (Xiao Y. et al., 2020). Figure 6D shows the composition of flora based on genus level, and LEfSe was used to compare the difference of microorganisms between each group and MC group (Figure 6E). The results showed that there were significant differences (*p* < 0.05) in 23 genera, including *Alloprevotella*, *Paramuribaculum*, *Adlercreutzia*, *Dwaynesavagella*, and *Paraprevotella*, in the intestinal flora of the five groups. Briefly, treatment interventions significantly altered the gut microbial structure of T2DM mice, where SV and DCHD interventions significantly reduced the relative abundance of *Dwaynesavagella* in T2DM mice, and DCHD-SV additionally significantly increased the relative abundance of *Paraprevotella* in T2DM mice. In a comparable context, it has been reported that the abundances of *Alloprevotella* and *Paraprevotella* are negatively correlated with the lipid profile in high fat diet-fed hamsters (Tong et al., 2020). It is known that *Paramuribaculum* is a *Muribaculum*-like genus within the family Muribaculaceae of the order Bacteroidales. The increasing of *Muribaculaceae* may contribute to enhancing the anti-diabetic properties of hypoglycemic drugs (Zhu et al., 2024). Furthermore, the abundance of *Adlercreutzia* in the diabetes-susceptible model is significantly lower than that in the non-susceptible model, which may serve as a potential intestinal flora marker for type 1 diabetes mellitus resistance (Rosell-Mases et al., 2023). These findings suggest that DCHD-SV may improve the symptoms of T2DM mice by regulating the intestinal flora, and *Dwaynesavagella* and *Paraprevotella* may serve as key bacterial taxa mediating the interventive effects of DCHD-SV in T2DM.

### 3.6 Correlations between intestinal flora and biochemical indicators

The Spearman correlation analysis was performed to assess the correlations between 23 differential bacterial genera in intestinal flora and biochemical indexes, and network diagram were plotted for data |r|>0.5 and *p* < 0.05 (Figures 6F,G). As shown in the figures, bacterial genera such as *Acutalibacter*, *Dwaynesavagella*, and *Prevotella* were positively correlated with LDL-c, TC, AUC of OTGG, HOMA-IRI, Liver TC, GSP, Liver LDL, and TG, and negatively correlated with HDL-c, HOMA-β, HOMA-ISI, QUICKI, and Liver HDL-c. In contrast, genera including UBA644, *Paramuribaculum*, *Adlercreutzia*, *Phocea*, and *Paraprevotella* were positively correlated with HDL-c, HOMA-β, HOMA-ISI, and QUICKI, and negatively correlated with LDL-c, TC, AUC of OGTT, HOMA-IRI, Liver TC, GSP, Liver LDL, and TG. In a previous study, *Dwaynesavagella* was confirmed to be positively correlated with liver LDL-c, TC, as well as serum TG, GSP, LDL-c and HOMA-IRI in T2DM mice (Yin et al., 2024), which is consistent with the findings of our present study. Additionally, it has been reported that *Paraprevotella* is negatively correlated with serum LDL-c in rats with lipid metabolism disorders, which also aligns with our findings (Li et al., 2019). The Spearman correlation analysis further revealed the relationship between intestinal flora and biochemical indicators, and suggested that DCHD-SV may improve insulin sensitivity and alleviate lipid metabolic disorders in T2DM mice by modulating the intestinal flora.

### 3.7 Effects of DCHD-SV on liver metabolites

**FIGURE 7:**
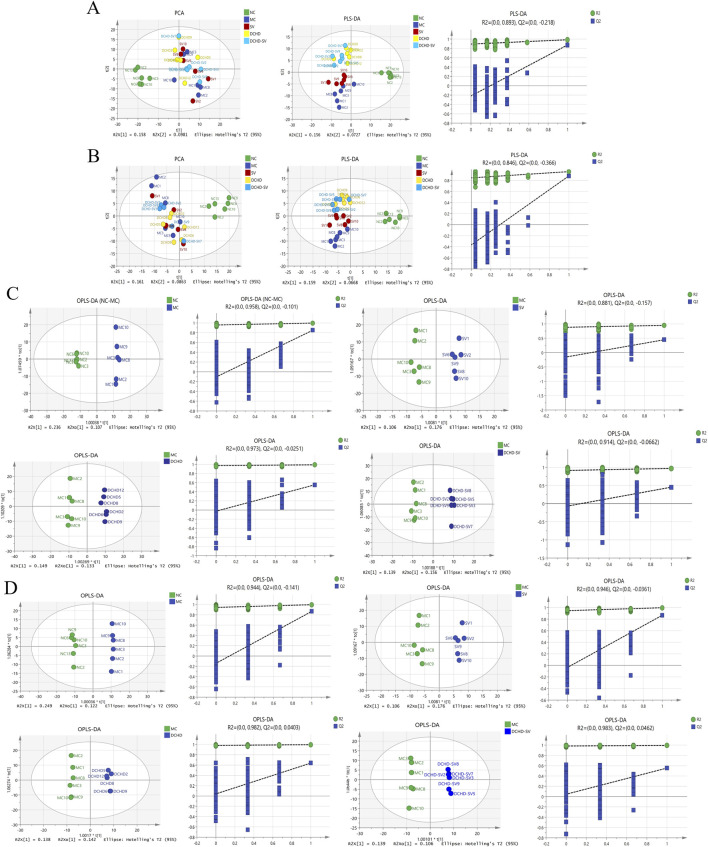
Multivariate statistical analysis of the liver metabolites in mice of each group (*n* = 6). The results of PCA, PLS-DA analysis and permutation tests for liver metabolites under positive **(A)** and negative ion modes **(B)**, and the results of OPLS-DA analysis and permutation tests between each group and MC group under positive **(C)** and negative ion modes **(D)**.

**FIGURE 8:**
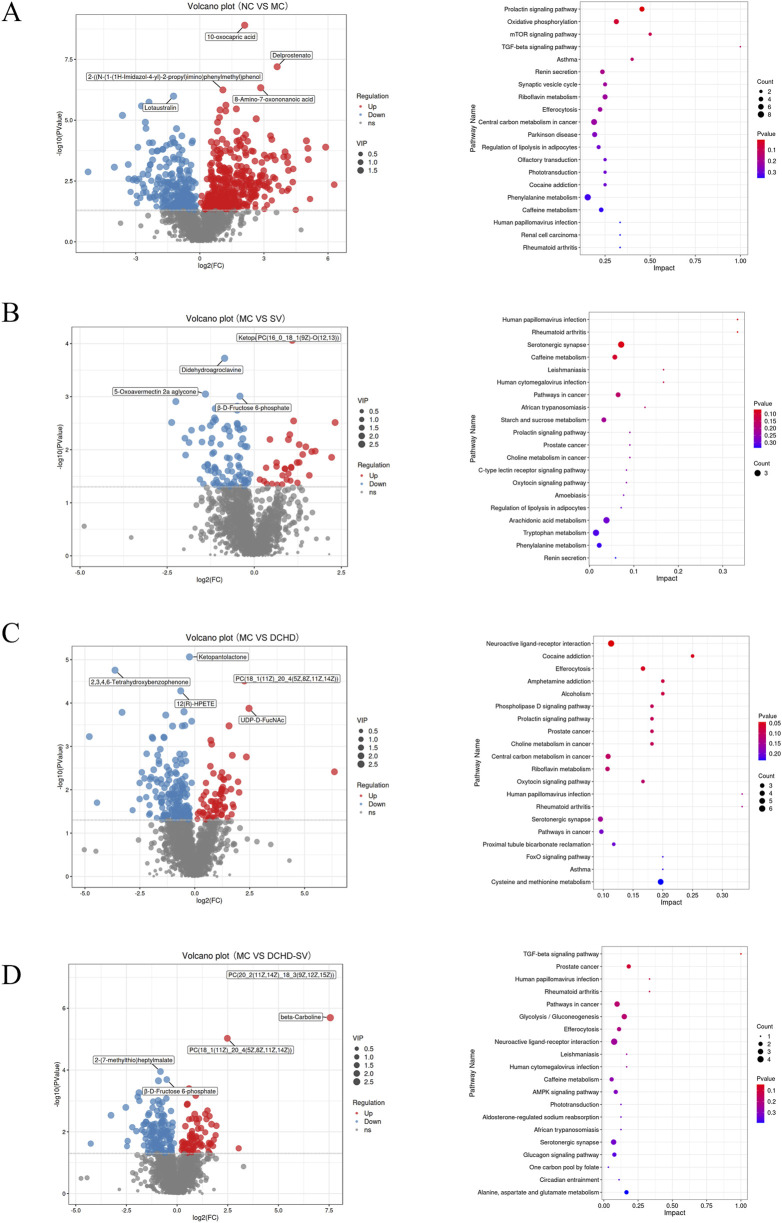
Effects of SV, DCHD, DCHD-SV administration on liver metabolites (*n* = 6). The significant differences in metabolites between MC group and NC **(A)**, SV **(B)**, DCHD **(C)**, DCHD-SV **(D)** groups, as well as the signaling pathways identified via KEGG enrichment analysis.

Metabolomics can help identify potential therapeutic targets, and improve the prevention and management of T2DM and its complications (Jin and Ma, 2021). The results of PCA, PLS-DA analysis and permutation tests for mice liver metabolites are shown in Figures 7A,B, and the OPLS-DA analysis and permutation tests between each group and MC group are shown in Figures 7C,D. These analyses revealed that T2DM significantly altered the liver metabolic profile of normal mice, whereas that of T2DM mice was significantly changed by different administration groups. Figures 8A–D displays the significant differences in metabolites and the signaling pathways identified via KEGG enrichment analysis. Compared with the NC group, the MC group significantly upregulated 386 metabolites and downregulated 253 metabolites. The KEGG enrichment showed that T2DM mainly affected the prolactin signaling pathway, oxidative phosphorylation, mTOR signaling pathway in mice. Additionally, compared with MC group, DCHD-SV group significantly upregulated 79 metabolites, including beta-carboline, PC (20 (11Z, 14Z) (9Z, 12Z, 15Z), PC (18 (11Z) (5Z, 8Z, 11Z, 14Z)), and downregulated 148 metabolites including 2-(7-methylthio)heptylmalate, beta-D-fructose 6-phosphate, and sulfadoxine. According to the results of KEGG enrichment, DCHD-SV mainly affected TGF-beta signaling pathway, pathways in cancer, glycolysis/gluconeogenesis signaling pathway and AMPK signaling pathway. AMPK can promote autophagy by inhibiting the mTOR pathway that directly dephosphorylates and inhibits downstream 70-kD ribosomal protein S6 kinase, so AMPK/mTOR-mediated autophagy may be an ideal target for the treatment of diabetic liver injury in T2DM (Zhu et al., 2021). As a key target identified by network pharmacology, activated PPARγ holds the potential to enhance insulin sensitivity, prevent lipid accumulation, improve glucose and lipid metabolism, and alleviate inflammatory damage simultaneously (Gong et al., 2024). Based on these, DCHD-SV may regulate T2DM by modulating the PPARγ/AMPK/mTOR signaling pathway, and further investigations into this mechanism will be conducted in our future study.

The differential metabolites (VIP>1, *p* < 0.05) between each group and MC group were crossed to get 21 biomarkers as shown in Supplementary Table S2, including FADH, spermidine, (+)gallocatechin, androstenedione and prostaglandin E2. Studies have shown that the reduction of FADH, potentially induced by metabolic disorders, oxidative stress, diseases, and genetic factors, can disrupt cellular energy metabolism and redox homeostasis (Abrosimov et al., 2024). It has been reported that supplementation of spermidine reduces receptor-interacting serine/threonine-protein kinase 1 (RIPK1)-mediated cell death, as well as insulin resistance and diabetic nephropathy induced by N-acetyltransferase 1 deficiency *in vivo* (Zhang et al., 2024). Gallocatechin significantly reduces FBG in DM rats, prevents BW loss, and alleviates oxidative stress in the skin of DM rat wounds by decreasing malondialdehyde content and increasing the levels of antioxidant enzymes (Vendidandala et al., 2021). Studies have demonstrated that androstenedione intervention had no effect on the proliferation and differentiation of human SGBS preadipocytes, but significantly increased TG accumulation in mature adipocytes (Ernst et al., 2020). Prostaglandin E2 prevents diabetic coronary atherosclerosis by stimulating M2 macrophage polarization through activating the CREB/BDNF/TrkB signaling pathway (Bi et al., 2020). These findings suggest that DCHD-SV may exert its anti-diabetic effect by regulating the levels of these biomarkers. Therefore, we aimed to further analyze the associations between these biomarkers, biochemical indicators, and intestinal flora via Spearman analysis.

### 3.8 Correlations of liver metabolites with biochemical indicators and intestinal flora

**FIGURE 9:**
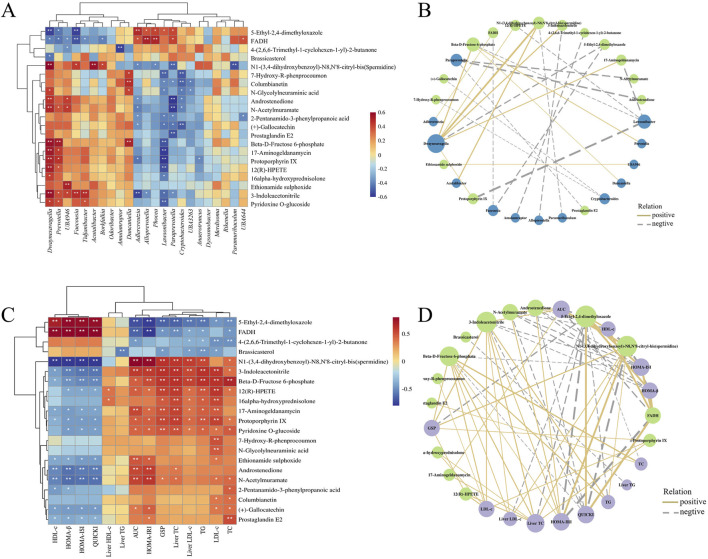
The heatmap of Spearman’s correlation analysis between the liver metabolites and biochemical indexes **(A)**, intestinal flora **(C)**, and their network plot **(B,D)** based on the correlations.

The correlations and network plots between 21 biomarkers in the mouse liver and biochemical indexes are shown in Figures 9A,B. The 5-ethyl-2,4-dimethyloxazole and FADH were positively correlated with HDL-c, HOMA-β, HOMA-ISI, and QUICKI, but negatively correlated with AUC of OGTT, HOMA-IRI, GSP, Liver TC, Liver LDL-c, TG, LDL-c, and TC, along with 4-(2,6,6-Trimethyl-1-cyclohexen-1-yl)-2-butanone, and Brassicasterol. And spermidine, 3-indoleacetonitrile, beta-D-fructose 6-phosphate, 17-aminogeldanamycin, protoporphyrin IX, ethionamide sulphoxide, androstenedione, N-acetylmuramate, (+)-gallocatechin, and prostaglandin E2 were negatively correlated with HDL-c, HOMA-β, HOMA-ISI, and QUICKI; in contrast, spermidine, 3-indoleacetonitrile, beta-D-fructose 6-phosphate, 12(R)-HPETE, 17-aminogeldanamycin, protoporphyrin IX, and pyridoxine O-glucoside were positively correlated with AUC, HOMA-IRI, GSP, Liver TC, Liver LDL-c, TG, LDL-c, and TC. It was reported that FBG level and HOMA-IRI were significantly increased in NAFLD mice, but decreased after supplementation with spermidine (Ni et al., 2022). Furthermore, in patients with polycystic ovary syndrome, there is a significant negative correlation between the androstenedione/free testosterone ratio and the area under the insulin response curve, insulin resistance, as well as the TC/HDL-c ratio (Lerchbaum et al., 2014). These results indicate that drug intervention can increase HDL-c, HOMA-β, HOMA-ISI, and QUICKI while decrease AUC of OGTT, HOMA-IRI, GSP, Liver TC, Liver LDL-c, TG, LDL-c, and TC in T2DM mice by regulating multiple liver metabolites.

The correlations and network plots between 21 biomarkers in the mouse liver and 23 differential genera in the intestinal flora are shown in Figures 9C,D. Bacterial genera such as *Dwaynesavagella*, *Prevotella*, and *Faecousia* were negatively correlated with 5-ethyl-2,4-dimethyloxazole and FADH, and positively correlated with compounds including spermidine, androstenedione, beta-D-fructose 6-phosphate, N-acetylmuramate, 12(R)-HPETE, 3-indoleacetonitrile, protoporphyrin IX, and 17-aminogeldanamycin. In contrast, genera like *Alloprevotella*, *Adlercreutzia*, *Lawsonibacter*, *Paraprevotella*, and *Cryptobacteroides* were positively correlated with 5-ethyl-2,4-dimethyloxazole and FADH, and negatively correlated with compounds such as 12(R)-HPETE, spermidine, 7-hydroxy-R-phenprocoumon, columbianetin, androstenedione, N-acetylmuramate, beta-D-fructose 6-phosphate, 17-aminogeldanamycin, protoporphyrin IX, and 3-indoleacetonitrile. These findings suggest that different administration groups may modulate the liver metabolic profiles of T2DM mice by altering the intestinal flora structure. Nevertheless, it is crucial to acknowledge that these correlative findings do not equate to causal relationships, and our mechanistic interpretations are subject to several limitations that need to be addressed in future studies. Meanwhile, although the mice data in this study provide valuable clues for the anti-diabetic effects of DCHD-SV, there are inherent physiological differences between mice and humans, which will limit the translation of our research findings to human clinical settings.

## 4 Conclusion

In this study, we investigated the therapeutic effects of SV, DCHD, and DCHD-SV on T2DM mice, as well as their impacts on intestinal flora and liver metabolites. The results have shown that SV, DCHD, DCHD-SV can effectively reduce fasting blood glucose, alleviate insulin resistance and hyperlipidemia symptoms in T2DM mice. Among them, the effects of DCHD-SV were better and more comprehensive than that of DCHD. Additionally, histopathological sections revealed that DCHD-SV also exhibited stronger restorative effects on liver, islet, and cecum injuries than DCHD. Meanwhile, the intervention of DCHD-SV modulated the intestinal flora and liver metabolic profiles in T2DM mice. The Spearman’s correlation analysis revealed that DCHD-SV might modulate tliver metabolites by modulating the abundances of *Dwaynesavagella*, *Acutalibacter*, *Prevotella* and *Paraprevotella*, thereby improving the abnormalities in biochemical indicators and the morphological damage of organ tissues in T2DM mice. Based on the results of network pharmacology and liver metabolomics, we propose that DCHD-SV may exert its anti-diabetic effects by activating the PPARγ/AMPK/mTOR pathway, which will be further investigated in our future studies.

## Data Availability

The datasets presented in this study can be found in online repositories. The names of the repository/repositories and accession number(s) can be found in the article/Supplementary Material.
