# Supplementary material for: Anti-diabetic effects of Dachaihu decoction combined with Sanghuangporus vaninii: insights from network pharmacology, intestinal flora, and liver metabolomics
Source: Front Cell Dev Biol. 2025 Nov 10;13:1707392. doi: 10.3389/fcell.2025.1707392 (PMC12640936; doi:10.3389/fcell.2025.1707392)
Supplement: Supplementary file 2 [file DataSheet1.docx]

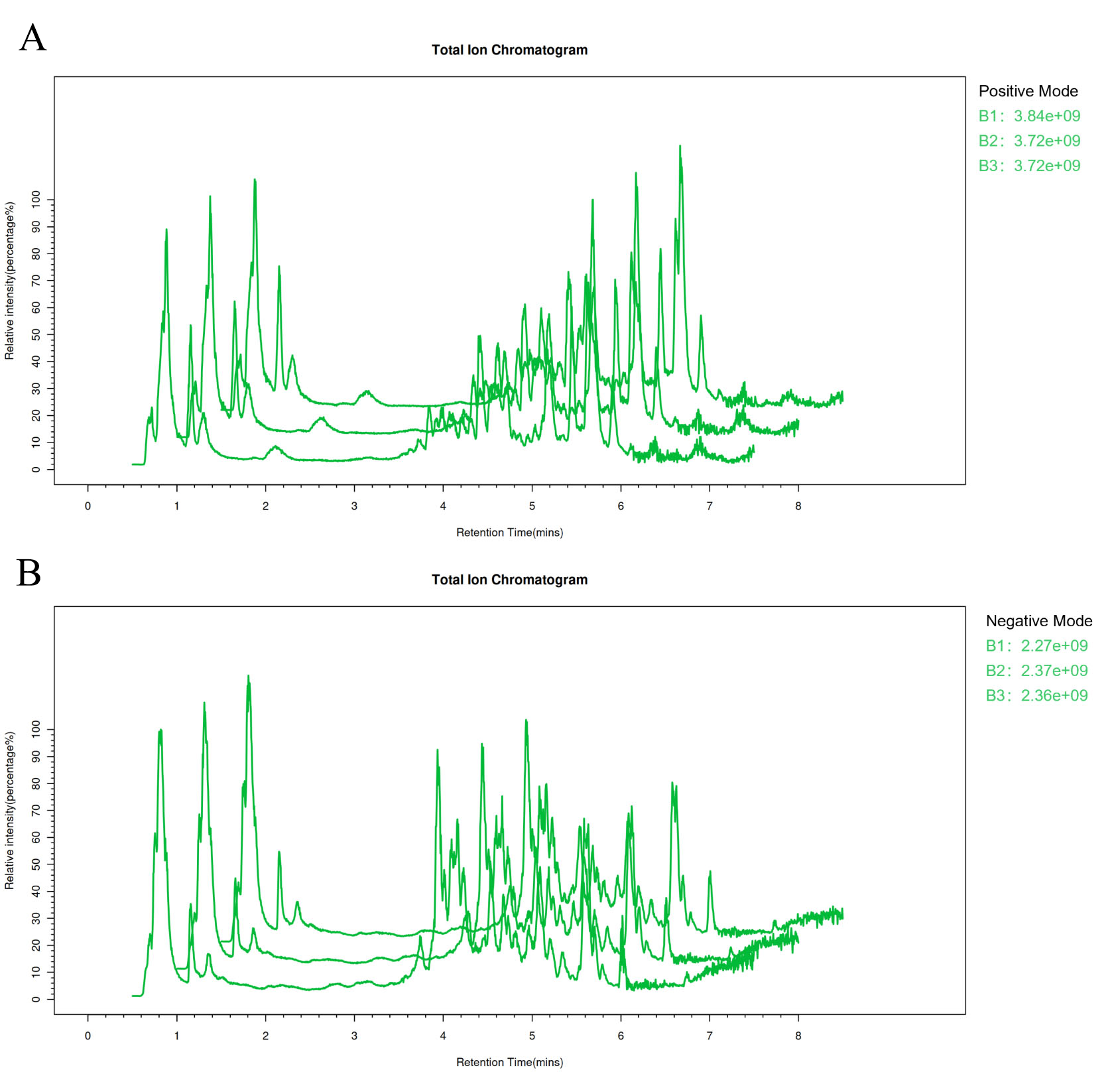


Figure S1. Total ion chromatography of DCHD-SV in positive (A) and negative ion (B) modes by UPLC-MS/MS.


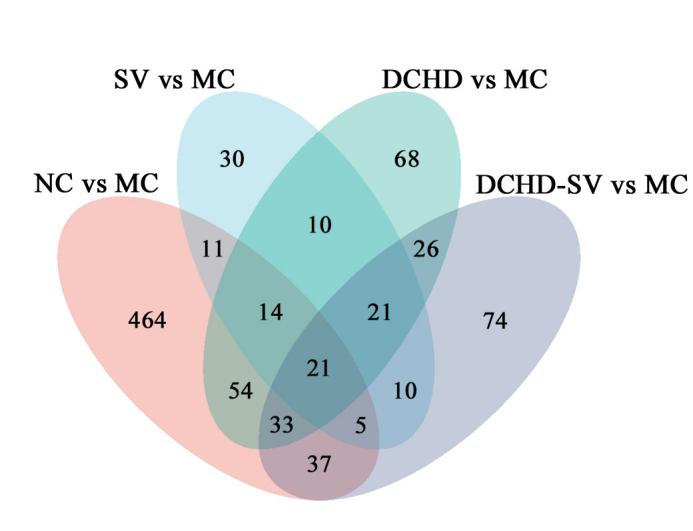


Figure S2. Venn diagram of differential metabolites among each comparison group.


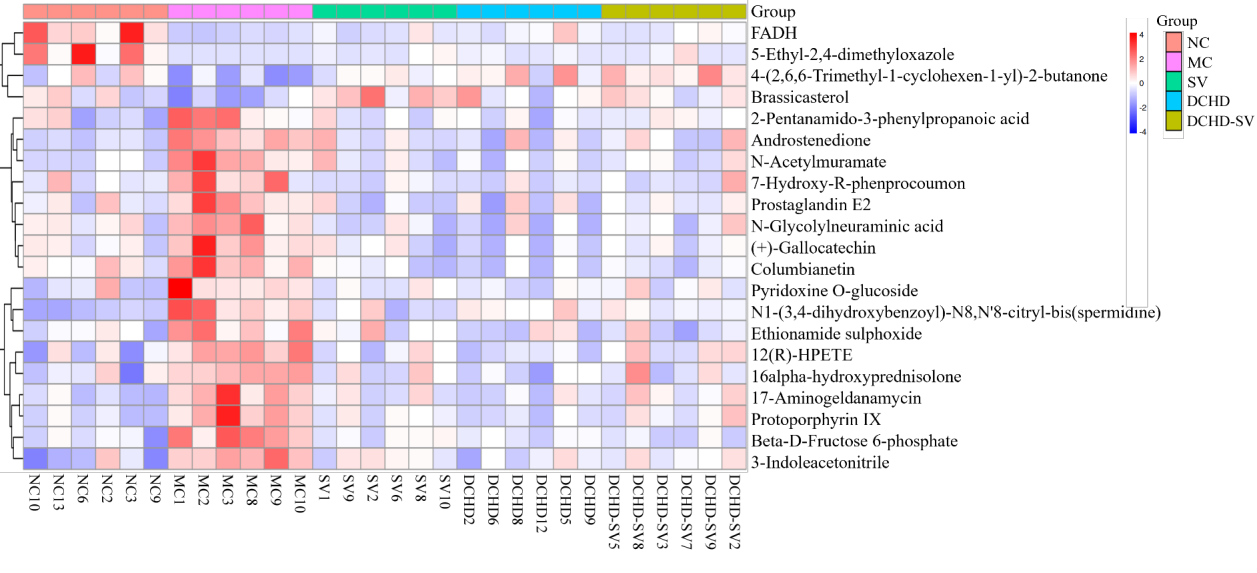


Figure S3. The hierarchical clustering heatmap of biomarkers.
